# Atypical findings in unilateral Acute Idiopathic Maculopathy: a case report

**Published:** 2020

**Authors:** Ayman Elnahry

**Affiliations:** * Department of Ophthalmology, Faculty of Medicine, Cairo University, Cairo, Egypt; Ophthalmic Diagnostic and Laser Unit, El Manial Hospital, Cairo University, Cairo, Egypt; Department of Ophthalmology, El Haram Hospital, Ministry of Health and Population, Giza, Egypt; Elnahry Eye Clinics, Giza, Egypt

**Keywords:** acute idiopathic maculopathy, atypical findings, cystoid macular edema, fluorescein angiography, intraretinal exudates, intraretinal hemorrhages, optical coherence tomography

## Abstract

Acute idiopathic maculopathy (AIM) is a condition reported to occur in young adults, which is classically characterized by the development of a unilateral serous macular detachment that commonly follows a flu-like illness. A 27-year-old female patient complained of diminution of vision in her left eye, of acute onset and progressive course for 2 weeks. She had a history of a flu-like condition 2 weeks prior. Following clinical examination, multimodal imaging analysis, and laboratory investigations, she was diagnosed with unilateral AIM, associated with atypical findings that were not previously reported to occur with AIM, including cystoid macular edema, flame shaped retinal hemorrhages, extensive hard exudates, and juxta-foveal yellowish intraretinal lesions. This may have been due to the more severe inflammation and concomitant phlebitis that was associated with this case compared to previously reported AIM cases.

## Introduction

Acute idiopathic maculopathy is a condition reported to occur in young adults and is classically characterized by the development of a unilateral serous macular detachment that commonly follows a flu-like illness [**1**]. It is suspected to follow infection by Coxsackievirus in some reported cases [**2**].

The condition was initially described as a unilateral neurosensory detachment associated with thickening of the underlying layer of retinal pigment epithelium, with vitreous cells and intraretinal hemorrhages occurring in some patients. However, these manifestations usually spontaneously disappear with no recurrence. Fluorescein angiography typically shows early hypofluorescent and hyperfluorescent areas with late increase in hyperfluorescence and associated dye pooling [**1**].

Later, new features associated with acute idiopathic maculopathy were reported, including papillitis with prominent veins and mild phlebitis, eccentricity of lesions, the association with pregnancy, peculiar subretinal exudation, and bilateral affection [**3**,**4**]. 

An atypical case of rare unilateral acute idiopathic maculopathy was reported, who, in addition to having a neurosensory detachment, had cystoid macular edema, flame shaped intraretinal hemorrhages, peculiar intraretinal material, and subretinal exudates. Fluorescein angiography images and unique optical coherence tomography findings were also presented.

## Case Report

A 27-year-old woman presented with decrease of vision in her left eye, of acute onset and progressive course for 2 weeks. She had a history of a flu-like condition 2 weeks prior. She had no other history of any systemic condition.

**Fig. 1:**
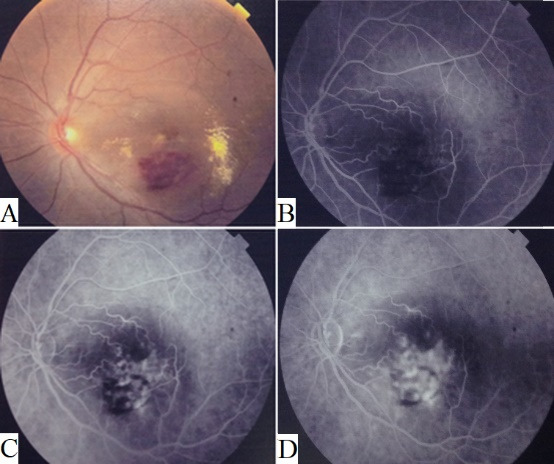
Clinical and fluorescein angiographic findings at presentation **A.** Color fundus photograph of the left retina showing cystoid edema of the macula, neurosensory detachment, retinal flame shaped hemorrhages, lipid exudates, and two juxta-foveal yellowish lesions located nasal and temporal to the fovea. **B.** Early phase of fluorescein angiography showing blocked fluorescence by retinal hemorrhages with mild patchy leakage. **C.** Late phase fluorescein angiography showing excessive leakage at the retinal pigment epithelium level under the area of retinal hemorrhages. **D.** Very late phase fluorescein angiography showing increased leakage and pooling of dye in the subretinal space

Examination revealed a best corrected visual acuity of 20/ 400 in her left eye and 20/ 20 in the right one. Anterior segment exam using slit lamp was normal in both eyes. Examination of the left macula revealed cystoid edema of the macula, neurosensory detachment, flame shaped retinal hemorrhages inferior to her fovea, hard exudates at the edges of the neurosensory detachment, and two juxta-foveal yellowish peculiar lesions, nasal and temporal, to the fovea (**Fig. 1a**). Examination of the periphery of the retina was unremarkable. Posterior segment exam of her right eye was completely normal.

 Fluorescein angiography of the left fundus was done and showed blockage of background fluorescence by the superficial retinal hemorrhages with patchy areas of mild hyperfluorescence in the early phase (**Fig. 1b**). Diffuse fluorescein leakage that was present at the level of the retinal pigment epithelium under the area of retinal hemorrhages appeared in the late phase (**Fig. 1c**) and increased in the very late phase (**Fig. 1d**), with dye pooling also evident in the subretinal space.

**Fig. 2:**
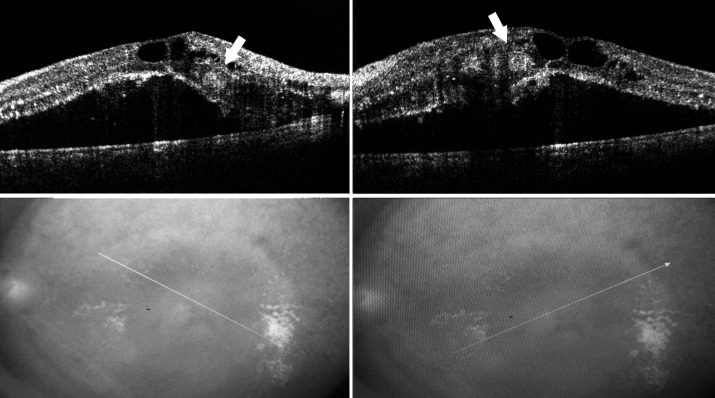
Optical coherence tomography imaging and scanning laser ophthalmoscopy at presentation. Optical coherence tomography imaging of the macular area showing cystoid macular edema, cystic spaces involving different retinal layers, neurosensory detachment, hard exudates, and 2 hyperreflective juxta-foveal globular peculiar lesions in the outer retina (white arrows), nasal and temporal to the fovea, that correspond to the two juxta-foveal yellowish lesions seen on fundus examination (Top right and left). Scanning laser ophthalmoscopic photography showing location of scans done (Bottom right and left)

 Optical coherence tomography imaging of the macular area revealed cystoid macular edema, cystic spaces involving both the outer and inner layers of the retina, neurosensory detachment, and 2 hyperreflective juxta-foveal globular lesions visible in the outer retina, which were nasal and temporal to the fovea, that corresponded to the two juxta-foveal yellowish peculiar lesions seen on fundus examination. There was no evidence of a choroidal neovascularization (**Fig. 2**). 

Laboratory investigations including complete blood picture, erythrocyte sedimentation rate, C-reactive protein, tuberculin skin testing, angiotensin converting enzyme level, rapid plasma reagin, antinuclear antibodies, and IgM antibodies against Bartonella and Toxoplasma, were all negative.

The condition of the patient gradually improved over several months on low dose systemic prednisone treatment with evidence of residual macular scarring and persistent moderate impairment of vision at the final follow-up visit.

## Discussion

This case represented a relatively more severe form of acute idiopathic maculopathy than previously reported cases [**1**-**3**], characterized by cystoid edema of the macula, subretinal fluid, flame-shaped hemorrhages, and subretinal exudation. This may have been due to a more severe inflammation caused by a different organism or an exaggerated host immune response.

The differential diagnosis included a branch retinal vein occlusion or an idiopathic choroidal neovascular membrane, which were both excluded by fundus fluorescein angiography imaging and optical coherence tomography, with findings more suggestive of an acute idiopathic maculopathy. Other conditions that might mimic acute idiopathic maculopathy also include central serous chorioretinopathy, however it is not usually associated with retinal hemorrhages as in the reported case.

The presence of more severe manifestations in the reported case may have been due to the occurrence of more severe phlebitis than has previously been reported in other cases of acute idiopathic maculopathy [**3**]. The associated phlebitis may have resulted in a picture similar to an occlusion of a retinal vein branch characterized by flame shaped hemorrhages in the retina with more severe leakage leading to the cystoid edema of the macula and subretinal exudation. The peculiar intraretinal material that was present nasally and temporally to the fovea was thought to represent the accumulation of inflammatory cells or debris as previously reported [**3**]. This material has also been previously reported to present as a pseudohypopyon or vitelliform material, which supports an inflammatory etiology for the condition [**5**,**6**].

In conclusion, a unilateral acute idiopathic maculopathy case associated with more severe features than previously reported, including cystoid edema of the macula, flame shaped hemorrhages in the retina, and subretinal exudation, was reported. Depicting the unusual and rare features associated with a condition is necessary to help avoid misdiagnosis and unnecessary treatment as has been previously reported in cases of acute idiopathic maculopathy [**7**].

**Consent for publication**

The patient signed a consent form for publication of her images.

**Conflict of interests**

The author declared that she has no competing interests.

**Funding**

No sources of funding were available.

**CARE guidelines**

This study adheres to CARE guidelines for reporting case reports.

**Acknowledgements**

None.

**Ethical approval**

This report was approved by Cairo University research ethics committee and followed the tenets of the Declaration of Helsinki.

## References

[1] Yannuzzi LA, Jampol LM, Rabb MF, Sorenson JA, Beyrer C, Wilcox LM Jr (1991). Unilateral acute idiopathic maculopathy. Arch Ophthalmol.

[2] Beck AP, Jampol LM, Glaser DA, Pollack JS (2004). Is coxsackievirus the cause of unilateral acute idiopathic maculopathy?. Arch Ophthalmol.

[3] Freund KB, Yannuzzi LA, Barile GR, Spaide RF, Milewski SA, Guyer DR (1996). The expanding clinical spectrum of unilateral acute idiopathic maculopathy. Arch Ophthalmol.

[4] Nakazawa T, Yamaguchi K, Shimura M, Yoshida M, Yoshioka Y, Tamai M (2003). Clinical Features of Bilateral Acute Idiopathic Maculopathy. Jpn J Ophthalmol.

[5] Fish RH, Territo C, Anand R (1993). Pseudohypopyon in unilateral acute idiopathic maculopathy. Retina.

[6] Mathew MRK, Webb LA, Bennett HGB, Hammer HM (2002). Unilateral acute idiopathic maculopathy (UAIM) masquerading as Best’s disease. Eye.

[7] Hoang QV, Strauss DS, Pappas A, Freund KB (2012). Imaging in the Diagnosis and Management of Acute Idiopathic Maculopathy. Int Ophthalmol Clin.

